# Toscana Virus Diagnosis via a Point-of-Care Tool Based on Disposable Optical Fiber Biosensors

**DOI:** 10.1021/acs.analchem.6c01827

**Published:** 2026-06-15

**Authors:** Federica Passeggio, Carla Zannella, Francesco Arcadio, Chiara Marzano, Rosalba Pitruzzella, Luigi Zeni, Bianca Maria Nastri, Anna De Filippis, Sven Reiche, Cécile Baronti, Bruno Coutard, Maria Grazia Cusi, Massimiliano Galdiero, Giuseppe Portella, Nunzio Cennamo

**Affiliations:** † Department of Engineering, 556687 University of Campania Luigi Vanvitelli, 81031, Aversa, Italy; ‡ Department of Translational Medical Sciences, University of Naples Federico II, 80131, Naples, Italy; § Department of Woman, Child and General and Specialized Surgery, 556687 University of Campania Luigi Vanvitelli, 80138, Naples, Italy; ∥ Department of Experimental Animal Facilities and Biorisk Management, Friedrich-Loeffler-Institut, 17493, Greifswald − Insel Riems, Germany; ⊥ Unité des Virus Émergents, Aix-Marseille Univ, Università di Corsica, IRD 190, Inserm 1207, IRBA, 13005 Marseille, France; # Department of Medical Biotechnologies, University of Siena, 53100, Siena, Italy; ∇ UOC Virology and Microbiology, University Hospital Luigi Vanvitelli, 80138, Naples, Italy

## Abstract

The Toscana virus (TOSV) is an emerging Phlebovirus endemic to the Mediterranean region, with marked neurotropism, responsible for often undiagnosed cases of meningitis and meningoencephalitis. Rapid and precise identification is critical for medical treatment, but traditional approaches like Polymerase Chain Reaction (PCR) and serology have limits in terms of time, cost, and potential cross-reactivity. This proof-of-concept introduces a low-cost point-of-care test (POCT) based on surface plasmon resonance (SPR) in plastic optical fibers (POFs) combined with an antibody selective for TOSV nucleoprotein. The POF biosensors were tested with serial dilutions of TOSV viral culture in phosphate-buffered solution (PBS), obtaining a detection limit of 0.78 PFU/mL and a highly reproducible dose–response curve. Reliable selectivity was demonstrated in testing with different viruses, including the measles virus, the herpes simplex type 1 virus, and the sandfly fever Naples virus. The POCT capability to measure viral titers was validated by evaluating clinical cerebrospinal fluid samples from infected patients together with PCR. The obtained results were consistent with PCR, suggesting approximately 3 orders of magnitude higher analytical sensitivity and demonstrating its potential as a useful POCT for improving TOSV infection surveillance and clinical management.

## Graphical abstract

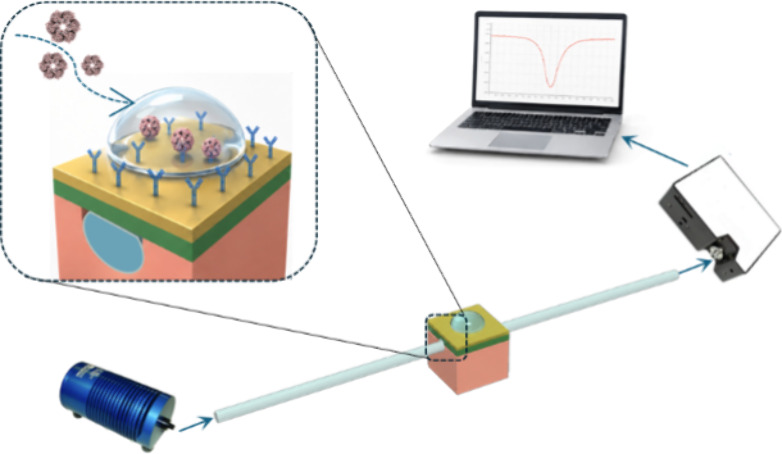

## Introduction

Toscana virus (TOSV) is an arbovirus belonging to the genus *Phlebovirus* (family *Phenuiviridae*) transmitted by phlebotomine sandflies. It was first identified in 1971 in central Italy.^1^ TOSV particles have a diameter of 80–120 nm^1^ and contain a negatively oriented, single-stranded RNA genome segmented into three parts: large (L), medium (M), and small (S). These segments encode RNA polymerase, two envelope glycoproteins (Gn and Gc), nucleoprotein (NP), and nonstructural proteins.^2^ Infection rates are higher during the summer months,^1^ coinciding with the peak abundance of its sandfly vectors.^3^ TOSV is one of three sandfly transmitted *Phlebovirus* serotypes, alongside the Naples and Sicilian serotypes. While Naples and Sicilian viruses are widely distributed due to the presence of the *Phlebotomus pappatasi* vector, TOSV has a more restricted geographic range.^2^ TOSV has been detected in several countries across Southern Europe (Italy, Spain, France, Portugal), the Eastern Mediterranean (Turkey, Cyprus), the Balkan Peninsula (Greece, Croatia, Bosnia and Herzegovina, Kosovo, Bulgaria), and North Africa (Tunisia, Morocco, Algeria). In Italy, the virus is endemic, with the highest number of cases reported in Tuscany and Emilia-Romagna. All confirmed cases are subject to mandatory reporting under the national health surveillance system, which is part of a broader plan for the prevention and control of arboviral infections.^3,4^ Despite its prevalence in Mediterranean regions, TOSV remains a scarcely known and underestimated pathogen.^5^ Although the animal reservoir remains unknown, anti-TOSV IgG has been detected in several species,^3^ while humans are considered terminal hosts.^3^

Most TOSV infections are asymptomatic or paucisymptomatic.^1^ However, due to its pronounced neurotropism,^1^ the virus can occasionally cause meningoencephalitis or encephalitis.^6^ Unlike the Sicilian and Naples viruses, which usually induce a febrile illness lasting several days,^6^ TOSV is a major cause of meningitis and meningoencephalitis in endemic regions.^1^ The spontaneous resolution of some infections without medical intervention contributes to significant underdiagnosis, underestimation, and under-reporting.^3^ Since the clinical symptoms of TOSV can be easily confused with those of other viruses and bacteria, rapid differential diagnosis is essential,^6^ not least to prevent the misuse of antibiotics.^7^ Currently, diagnosis primarily relies on Polymerase Chain Reaction (PCR) of cerebrospinal fluid (CSF), blood, and, less commonly, urine.^5^ Simultaneous detection of antigen-specific IgM and IgG antibodies is desirable; however, the reliability of serological tests is limited by potential cross-reactivity with cocirculating other viruses in certain regions^8^ and the lack of commercially available kits. Other diagnostic approaches include indirect immunofluorescence assay (IIFA), plaque reduction neutralization test, enzyme immunoassay (EIA), and immunoblotting (IB). These methods are technically demanding, time-consuming, and require specialized personnel and laboratories.^1^ Therefore, there is a pressing need to develop rapid diagnostic tests, such as point-of-care tests (POCTs), particularly in critical settings, such as emergency rooms, where timely identification of the causative agent is crucial for guiding therapeutic decisions.^9^

Biosensors are becoming increasingly important for the rapid and accurate detection of viruses and bacteria.^10^ Their high sensitivity and specificity, combined with fast response times, give them a clear advantage over traditional laboratory tests. These qualities make biosensors especially valuable in situations where time is critical, such as emergency care or environmental monitoring. A very interesting application is the integration of biosensors with plastic optical fibers (POFs). These fibers are notable for their flexibility, robustness, and low cost, making them ideal for developing portable diagnostic devices that can be used even in challenging environments or remote areas far from medical centers.^11^ POFs can be combined with molecular recognition elements, which may be biological, such as antibodies or aptamers, or synthetic, such as molecularly imprinted polymers.^11–13^ One of the most promising approaches in this direction is based on surface plasmon resonance (SPR), which exhibits extraordinary sensitivity to refractive index (RI) variations at the surface of the metallic nanofilm. In combination with selective receptors, SPR enables real-time monitoring of molecular interactions. Indeed, the use of plasmonic sensors for detecting pathogens, such as viruses and bacteria, has attracted increasing attention in recent years.^14,15^ Along this line, an SPR-POF biosensor was developed for the detection of respiratory syncytial virus (RSV) by exploiting an antibody targeting the viral fusion (F) protein. The RSV biosensor demonstrated a low limit of detection (LOD) of approximately 1 plaque-forming unit (PFU)/mL.^10^

As evidenced by ELISA and Western blot analyses, the immune response to TOSV is primarily directed against NP, the dominant antigen. Only one-third of the patients present antibodies against G1 and G2 surface antigens, indicating the lower immunogenicity of these epitopes. In addition, serological tests based on NP show greater specificity, allowing cross-reactivity with Neapolitan and Sicilian serotypes to be ruled out.^6^ Based on this evidence, using NP as the target for the developed biosensor appeared to be the most appropriate sensing strategy. Therefore, in this work, an antibody targeting the NP is used to achieve the biosensor. Although NP is commonly considered intraviral, there is abundant experimental evidence and indirect observations indicating that it can occasionally be exposed or released into the extracellular compartment for various reasons.^16–20^

Based on this evidence, this study presents a biosensor targeting the TOSV NP for rapid, sensitive diagnosis of infection. NP was selected as an antigenic target due to its high immunogenicity and specificity, as well as its documented presence in the extracellular compartment during infection. In more detail, in this work, first, preliminary experiments were conducted in phosphate-buffered saline (PBS) using serial TOSV concentrations to generate a dose–response curve with the proposed SPR biosensor. Then, selectivity was evaluated using viruses other than TOSV. Finally, real cerebrospinal fluid samples from TOSV-infected patients were tested together with the gold standard.

## Materials and Methods

### Chemicals

*N*-Hydroxysuccinimide (NHS), N-(3-(Dimethylamino)­propyl)-N′-ethylcarbodiimide hydrochloride (EDC), (±)-α-Lipoic acid, ethanolamine, and phosphate-buffered saline (PBS) were purchased from Merck KGaA (Darmstadt, Germany); purified monoclonal antibody ab (clone 8F2A1) against the TOSV N-protein obtained from the Friedrich-Loeffler-Institut (Greifswald – Insel Riems, Germany).

### Sensing Strategy and Theoretical Background

In this work, instead of the viral surface antigen,^10^ the NP is used as the target for biosensor development. Therefore, the receptor used is an anti-NP. Although NP is commonly considered intraviral, there is abundant experimental evidence and indirect observations indicating that it can occasionally be exposed to or released into the extracellular compartment and thus be detectable in biological samples even without artificial viral lysis. First of all, NP exposed on the cell surface or actively secreted: in some viruses, NP is actively transported on the cell surface or secreted, as in the case of measles virus, where NP can pass to late endosomes or interact with FcyR and be exposed or secreted.^16^ Later, spontaneous or immune system-induced cell lysis: some viruses, even “non-cytopathic” ones, can cause host cell death by apoptosis or necrosis, resulting in the release of internal proteins, including NP. For example, in mice infected with Lymphocytic Choriomeningitis Virus (LCMV), NP is released in serum and CSF, following lysis mediated by CD8^+^ T lymphocytes.^17^ At other times, however, infected cells can be directly destroyed by cytotoxic T lymphocytes, thereby releasing NP. For example, in the LCMV model, the amount of NP released correlates with Cytotoxic T Lymphocyte (CTL) activity, peaking during the effective phase of the CD8 response.^17^ Another reason regards environmental conditions: pH, temperature and oxidative stress. Adverse environmental conditions, such as changes in temperature and/or pH, oxidative stress, and uncontrolled freezing/thawing cycles, can compromise the integrity of the viral capsid, inducing partial or complete disassembly, conformational changes resulting in the exposure or release of internal proteins, such as NP. For example, in the case of Norwalk virus, at pH 8 and/or temperatures >60 °C, a reversible dissociation of the capsid with release of internal proteins is observed.^18^ Other examples include the Influenza A virus, in which an acidic pH (pH 4) for just 30 s causes loss of virion integrity,^19^ and Adeno-Associated Virus (AAV8), in which analysis by mass spectrometry shows that the capsid destabilizes as early as 55–65 °C.^20^ In addition, TOSV NP has been used effectively as a recombinant antigen in ELISA assays to detect IgM and IgG antibodies in the serum of infected patients.^1^

### Antibody: Provenance, Production and Purification

A total of 50 mL of TOSV strain UVE/TOSV/2016/FR/6371 (EVA ref-SKU: 001 V-02462) was produced by infecting Vero cells cultured under serum-free conditions, without fetal bovine serum (FBS). Cell culture supernatants containing virus were clarified by two consecutive centrifugations at 4000 × g for 10 min at 4 °C to remove cellular debris. Viral inactivation was performed by adding 2 mL of a 5% formaldehyde working solution (pH 7.0) to the clarified supernatants, yielding a final concentration of 0.2% formaldehyde. The mixtures were incubated at 30 °C for 1 h in a dry water bath. When immediate downstream processing was not possible, samples were stored at 4 °C. Following inactivation, the viral supernatants were concentrated using Amicon Ultra-15 centrifugal filter units (Merck Millipore) according to the manufacturer’s instructions, until a final volume of 500 μL was obtained. The concentrated samples were then aliquoted into five 100 μL portions and stored at – 80 °C until further use.

BALB/c mice were immunized intraperitoneally five times at four-week intervals with 100 μL of inactivated, purified TOSV mixed with an equal amount of GERBU Adjuvant MM (GERBU Biotechnik GmbH). Four days after the final boost, the immunized mice were euthanized, and the spleens were removed under aseptic conditions. Finally, mouse spleen cells were fused with SP2/0 myeloma cells as previously described,^21^ and the resulting hybridoma cells were assessed for mAb against TOSV by indirect immunofluorescence and Western blotting. Purification of mAb was performed using HiTrap Protein G antibody purification columns according to the manufacturer’s instructions (Fisher Scientific, Germany).

### Cells and Viruses Preparation

Vero cells (ATCC CCL-81, Manassas, VA, USA) were maintained in Dulbecco’s Modified Eagle Medium (DMEM; Microtech, Naples, Italy) supplemented with 4.5 g/L glucose, 10% FBS (Microtech), and 1% penicillin-streptomycin solution (Himedia, Naples, Italy). Vero/hSLAM cells (ECACC 04091501, Porton Down, UK) were cultured in the same conditions with the addition of Geneticin (G418, Sigma-Aldrich, St. Louis, Missouri, United States).

Vero cells were infected with TOSV (strain ISS Phl.3, European Virus Archive [EVAg], Marseille, France), herpes simplex virus type 1 (HSV-1; strain SC16), and sandfly fever Naples virus (SFNV; strain UVE/SFNV/UNK/IT/30451, EVAg) at a multiplicity of infection (MOI) of 0.1. Vero/hSLAM cells were used to propagate measles virus (MeV; strain Edmonston, ATCC VR-24) at the MOI of 0.1, as previously described.^22^

Infected cells were harvested upon the appearance of virus-induced cytopathic effects (CPE), and viral titers were determined by plaque assay. The resulting stock titers were 1 × 10^10^ PFU/mL for TOSV and HSV-1, 1 × 10^8^ PFU/mL for MeV, and 1 × 10^7^ PFU/mL for SFNV. Viruses were concentrated by using the Intact Virus Precipitation Reagent (Invitrogen, Carlsbad, California, United States). A stock of TOSV was lysed using a RIPA buffer composed of 50 mM Tris-HCl (pH 7.4), 150 mM NaCl, 1% NP-40, 0.1% sodium dodecyl sulfate (SDS), 0.5 M ethylenediaminetetraacetic acid (EDTA), 1.5 mM dithiothreitol (DTT), and a protease inhibitor cocktail. The titer of the Red TOSV stock was 1 × 10^10^ PFU/mL.

### Western Blot

Cells (1 × 10^6^) were infected with each virus at the MOI of 0.1 and harvested at 24 h postinfection (hpi). Cells were lysed with RIPA buffer, and protein concentrations were determined. An equal amount of proteins (30 μg per sample) was separated by SDS-PAGE. Following electrophoresis, proteins were transferred onto polyvinylidene difluoride (PVDF) membranes (Millipore Corporation, Darmstadt, Germany) and blocked with 5% bovine serum albumin (BSA). Membranes were incubated with the following primary antibodies: anti-TOSV NP (1:100; 100P-01625, EVAg) and anti-GAPDH (1:10,000; E-AB-48016, Elabscience, Houston, TX, USA), followed by incubation with horseradish peroxidase (HRP)-conjugated goat antimouse IgG secondary antibody (Bio-Rad, Berkeley, CA, USA). Protein bands were visualized using enhanced chemiluminescence (ECL; Thermo Scientific, Rockford, IL, USA) on a ChemiDoc Imaging System (Bio-Rad, Hercules, CA, USA). Densitometric analysis of immunoreactive bands was performed using ImageLab software version 6.1 (Bio-Rad, Hercules, CA, USA).

### SPR-POF Chip

The SPR-POF chip was fabricated according to a well-established procedure reported in.^23^ The probe consists of a modified POF with an overall diameter of 1 mm and a poly­(methyl methacrylate) (PMMA) core with a RI of 1.49. The POF was embedded in a resin support and subsequently polished to remove the cladding and a portion of the core. In this way, a sensitive D-shaped area suitable for the following steps was created. A thin intermediate film of Microposit S1813 photoresist, approximately 1 μm thick and with an RI of 1.61, was spin-coated onto this sensitive region. This layer serves as both an optical buffer that enhances the coupling conditions for surface plasmon excitation and an adhesion promoter for the subsequent metallic coating.^23^ Finally, a 60 nm gold film was sputtered onto the photoresist layer.

### SPR-POF Biochip

To make the SPR-POF chip a biosensing platform capable of detecting the TOSV, a functionalization protocol was carried out to immobilize a specific antibody directed against the TOSV internal protein on the plasmonic surface. The functionalization process of the D-shaped sensitive area followed an established multistep procedure protocol.^10,24,25^ Initially, the gold-coated surface was rinsed three times with Milli-Q water for 5 min each to remove any impurity. The cleaned surface was then incubated in a 0.3 mM lipoic acid solution (prepared in 8% ethanol) for 18 h to generate carboxyl-terminated sites. Subsequently, these carboxyl groups were activated for 20 min using an NHS/EDC mixture (200 mM/50 mM in PBS, pH 7.4), followed by three washes with PBS. A 15 μL aliquot of anti-TOSV NP antibody (0.5 mg/mL) was then applied and incubated for 2 h to enable covalent binding to the activated surface. After antibody immobilization, unreacted carboxyl groups were blocked with 1 M ethanolamine solution (pH 8.0) for 30 min. The resulting SPR-POF biochip was stored in PBS at 4 °C overnight prior to use.

### Experimental Setup

The experimental configuration developed for testing the SPR-POF biochip was designed to be compact, low-cost, and easily integrable into a portable POCT device. The sensor system comprises a broadband halogen light source (HL-2000-LL, Ocean Optics, Orlando, FL, USA) providing an emission spectrum from 360 to 1700 nm, and a spectrometer (SR-6VN500, Ocean Optics, Orlando, FL, USA) operating within the 300–1000 nm detection range.^10,23^ The SPR-POF biosensor is optically coupled to a white-light source via a 50:50 POF-based optical splitter and to a spectrometer. All connections are performed using removable SMA connectors. The spectrometer is connected to a personal computer for data acquisition via OceanView software (version 2.0.16, Ocean Optics), and spectral analysis is subsequently performed using MATLAB (version R2022b, MathWorks, Natick, MA, USA). Figure 1 shows a picture of the POCT.

**Figure 1:**
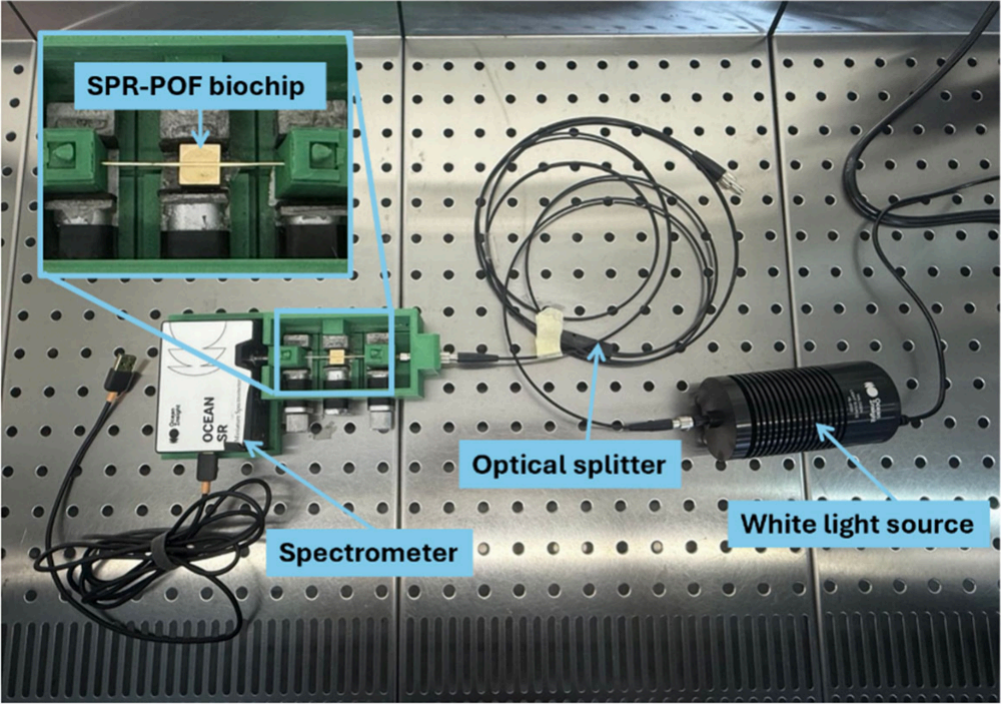
Experimental setup based on a white light source and a spectrometer to test the SPR-POF biochip.

### Sample Preparation and Measurement Protocol

To obtain the calibration curve, a TOSV culture in DMEM medium was used as the starting sample, with an initial concentration of 10^10^ PFU/mL. From this culture, serial dilutions in PBS at known concentrations were prepared and tested in order to construct the calibration curve.

For the initial tests, the serial dilutions obtained from the TOSV culture were used without any pretreatment. Subsequently, serial dilutions of the same culture were tested after undergoing a mild lysis pretreatment with RIPA buffer. To evaluate selectivity, cultures of different viruses were subsequently tested, which share similarities with TOSV in terms of structure and/or clinical manifestations. The viruses tested as interferents were HSV-1, MeV and SFNV. In this case, the viral cultures were subjected to serial dilutions in PBS. Given the difficulty of obtaining clinical samples positive for TOSV, clinical isolates derived from the cerebrospinal fluid of infected patients were used as a real sample to detect TOSV in a complex matrix. These isolates were propagated in vitro in Vero cells. Specifically, the samples included a blank sample (supernatant from uninfected cells), a positive sample (clinical isolates from the cerebrospinal fluid of infected patients), and a negative sample (TOSV-negative cerebrospinal fluid). In this case, the samples were subjected to serial dilutions in PBS.

To test the SPR-POF biochip, 40 μL of the sample solution was deposited onto the sensor’s sensitive surface for each measurement. The SPR spectra were obtained by normalizing the transmitted spectra to that acquired in air, which was considered as a reference spectrum, since the SPR condition is not satisfied in air.^23^ The sample was incubated at room temperature for 10 min. At the end of incubation, the surface was thoroughly washed with PBS and the resonance spectrum was then acquired with PBS as surrounding medium. The adoption of this PBS washing and acquisition protocol enabled the elimination of signal variations attributable to nonspecific binding to the sensor surface or to variations in the sample matrix (bulk effect), thereby isolating the signal derived from specific binding events.

### qPCR

On the day of infection, a series of dilutions of the four clinical isolates-derived samples was prepared. Viral RNA was isolated from the infected culture using TRIzol (Thermo Fisher Scientific, Waltham, MA, USA) and quantified via absorbance measurements on a NanoDrop 2000 spectrophotometer (Thermo Fisher Scientific). cDNA synthesis was carried out using the 5× All-In-One RT MasterMix (Applied Biological Materials, Richmond, Canada), and 2 μL of each sample served as the template for quantitative PCR (qPCR). Amplification targeted the TOSV NP protein gene (forward: GAGTTTG­CTTACCAA­GGGTTTG; reverse: AATCCTAA­TTCCCCTA­ACCCCC) and the TOSV *M* gene (forward: TCAATTC­AGCAAGTA­ACATAC­AATGG; reverse: CGTGGTC­TGTCTTGG­TTGATG) (PMID: 40732759). GAPDH (forward: CCTTTCA­TTGAGC­TCCAT; reverse: CGTACAT­GGGAG­CGTC) was used as an internal control to assess RNA integrity and efficiency of reverse transcription. The Ct values reported for TOSV NP and M genes correspond to target amplification signals and are presented without ΔCt transformation.

All primers were designed using Primer3 (version 4.1.0) and obtained from Eurofins (Ebersberg, Germany). qPCR measurements were performed in technical triplicates, and Ct values are reported as mean ± standard deviation.

## Results and Discussion

### NP Expression in Western Blot

To evaluate the binding specificity of the anti-TOSV NP antibody used in this study, we assessed NP expression in TOSV-infected cells and in cells infected with other encephalitis-causing viruses (HSV-1, MeV, and SFNV). Cells were infected with each virus, harvested, and lysed to extract proteins at 24 hpi. NP expression levels were analyzed by Western blot and normalized to GAPDH (Figure 2).

**Figure 2:**
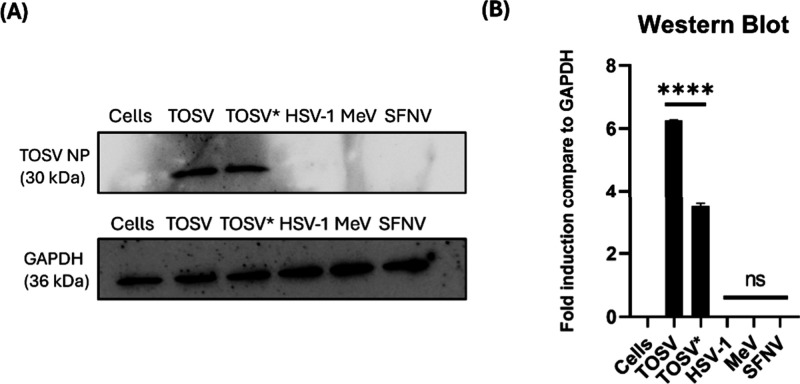
Detection of TOSV NP by Western blot. (A) Cell lysates from uninfected cells (lane 1), TOSV-infected cells (lane 2), and TOSV-infected cells after lysis treatment (indicated with *, lane 3) were analyzed alongside lysates from cells infected with unrelated viruses: HSV-1 (lane 4), MeV (lane 5), and SFNV (lane 6). The lower panel shows the loading control (GAPDH). (B) Band intensities of the TOSV NP were quantified and normalized to GAPDH, showing consistent expression levels between lanes 2 and 3.

As shown in Figure 2, a clear band corresponding to the TOSV NP was detected exclusively in TOSV-infected cells (lane 2) and in lysed TOSV-infected cells (lane 3), whereas no signal was observed in uninfected cells (lane 1) or cells infected with the other viruses, including HSV-1 (lane 4), MeV (lane 5), and SFNV (lane 6). The NP band was of consistent intensity in lanes 2 and 3, indicating that the lysis procedure did not affect protein detectability. The loading control (GAPDH) was uniformly expressed across all lanes, confirming equal protein loading. These results demonstrate the specificity of the antibody for TOSV NP and the absence of cross-reactivity with other tested viruses.

### TOSV Detection via POCTs

#### Plasmonic Characterization of SAM on SPR-POF Biochips

The successful receptor layer formation on the SPR-POF chip was assessed by monitoring the SPR spectra before and after the functionalization steps (detailed in paragraph SPR-POF biochip of Materials and Methods) with pure water as the surrounding medium.

As shown in Figure S1 in the Supporting Information, the surface modification resulted in a red shift (i.e., the resonance wavelength value increased) of approximately 11 nm in the resonance wavelength. The magnitude of this resonance wavelength shift is consistent with values reported for similar SPR-POF biosensors employing different molecular recognition elements.^26–28^

More specifically, it is important to recall that for the used SPR-POF probe, when the refractive index in contact with the SPR surface increases, the resonance wavelength shifts to longer wavelengths (a red shift). Therefore, the functionalization process induces a shift of the SPR peak toward higher wavelengths (red shift), since the immobilization of specific antibodies onto the gold SPR surface leads to the formation of a receptor layer at the gold–dielectric interface, increasing the refractive index with respect to the bare surface, when the same bulk solution (e.g., pure water) is present.

#### Binding Tests in PBS

In a first step, to evaluate the biosensor’s performance, binding assays were performed using TOSV culture without pretreatment in PBS at different concentrations. Figure 3A shows the SPR spectra obtained after incubating the biosensor with increasing concentrations of TOSV from 1 PFU/mL to 1000 PFU/mL. In particular, as the TOSV concentration increases, the resonance wavelength shifts to lower values (a blue shift). In particular, when the antibody on the functionalized surface binds the analyte (NP) during the interaction phase, this binding induces a conformational change in the bioreceptor layer, associated with a decrease in the refractive index in contact with the SPR sensing interface. This phenomenon ultimately results in a shift of the SPR peak toward lower wavelengths (blue shift). This behavior can be attributed to a more efficient conformational reorganization of the antibody following the antigen–antibody binding, and it was already observed in previous studies where the same SPR-POF transducer was coupled to different antibody-based receptors targeting a wide range of analytes having different molecular weights.^29^ Figure 3B shows the dose–response curve, which plots the absolute value of the resonance wavelength shift |Δλ| versus the TOSV concentration (c). The experimental results reported in Figure 3B were fitted using the Langmuir equation to obtain the dose–response curve. The Langmuir model is recalled below:

1 $$\left|{\Delta \lambda }_{c}\right|=\left|\lambda_{c}-\lambda_{0}\right|=\left|{\Delta \lambda }_{\max}\right|\cdot \frac{c}{K+c}$$

where *c* is the TOSV concentration, λ_c_ and λ_0_ are the resonance wavelengths, and Δλ_max_ is the maximum wavelength at the saturation value. In particular, λ_c_ is the resonance wavelength measured at concentration *c*, and λ_0_ is the resonance wavelength in the absence of the analyte (blank solution). K is the Langmuir fitting constant. Table S1 in the Supporting Information reports the Langmuir fitting parameters, obtained using OriginPro, relative to Figure 3B.

**Figure 3:**
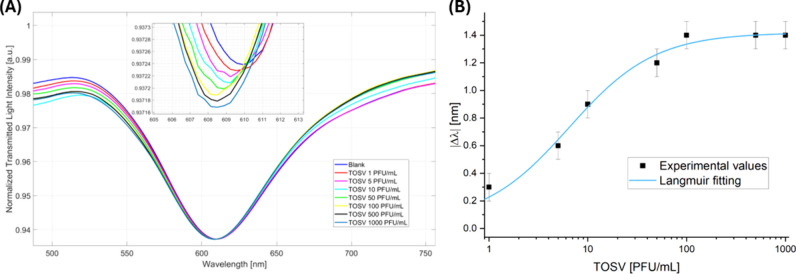
Detection of TOSV in PBS via POCT. (A) SPR spectra obtained using the SPR-POF biosensor with TOSV solutions at different concentrations (from 1 PFU/mL to 1000 PFU/mL) in PBS. (B) Dose–response curve for TOSV detection in PBS. The absolute variation in resonance wavelength (Δλ) relative to the blank as a function of viral concentration on a semilogarithmic scale. The black squares indicate the experimental data, while the dark cyan curve is the interpolation obtained using the Langmuir model. The error bar (equal to about 0.1 nm) represents the maximum standard deviation calculated on three sensors replicated under the same conditions.

To evaluate reproducibility, three similar SPR-POF biochips were developed and tested for TOSV detection. More specifically, the experimental values of the dose–response curve shown in Figure 3B represent the average of the resonance wavelength shifts obtained from these measurements. The error bars for the data points were calculated from three independent measurements performed under the same conditions, using three different biochips, and represent the maximum standard deviation (worst case). This value, equal to about 0.1 nm and reported in Figure 3B, is not used to estimate sensor performance; it is useful for assessing the quality of the model error, meaning the standard deviation of the blank in the Langmuir fitting used to estimate the LOD.

At very low concentrations of the analyte (i.e., *c* ≪ *K*, eq 1) can be considered linear, and the slope of this linear function (|Δλ_max_|/K) is defined as sensitivity at low concentrations (S_low c_). In this work, the low-concentration sensitivity (S_low c_) of the developed SPR-POF biosensor was found to be equal to 0.21 [(nm × mL)/ PFU]. The LOD is defined as 3.3 times the standard deviation of the blank signal (obtained from the Langmuir fitting parameters) divided by the low-concentration sensitivity (S_low c_). Therefore, the resulting limit of detection (LOD) was equal to 0.78 [PFU/mL].

As additional evidence, the lysed TOSV culture was tested. Specifically, several dilutions were prepared from a lysate containing 1 PFU/mL of TOSV. As shown in Figure 4, diluting 1 PFU/mL of lysed TOSV to high dilution ratios (1:10000, 1:2000, 1:1000, 1:200) resulted in a blue shift. Therefore, these results demonstrate a difference of approximately 3 orders of magnitude in the TOSV NP concentration between untreated and lysis-treated cultures. This result was reasonably expected, since the lysis treatment causes an increase in the concentration of free NP in the sample, making it more available for binding to the receptor layer immobilized on the sensing surface and, consequently, increasing in absolute value the refractive index variation of the receptor layer.

**Figure 4:**
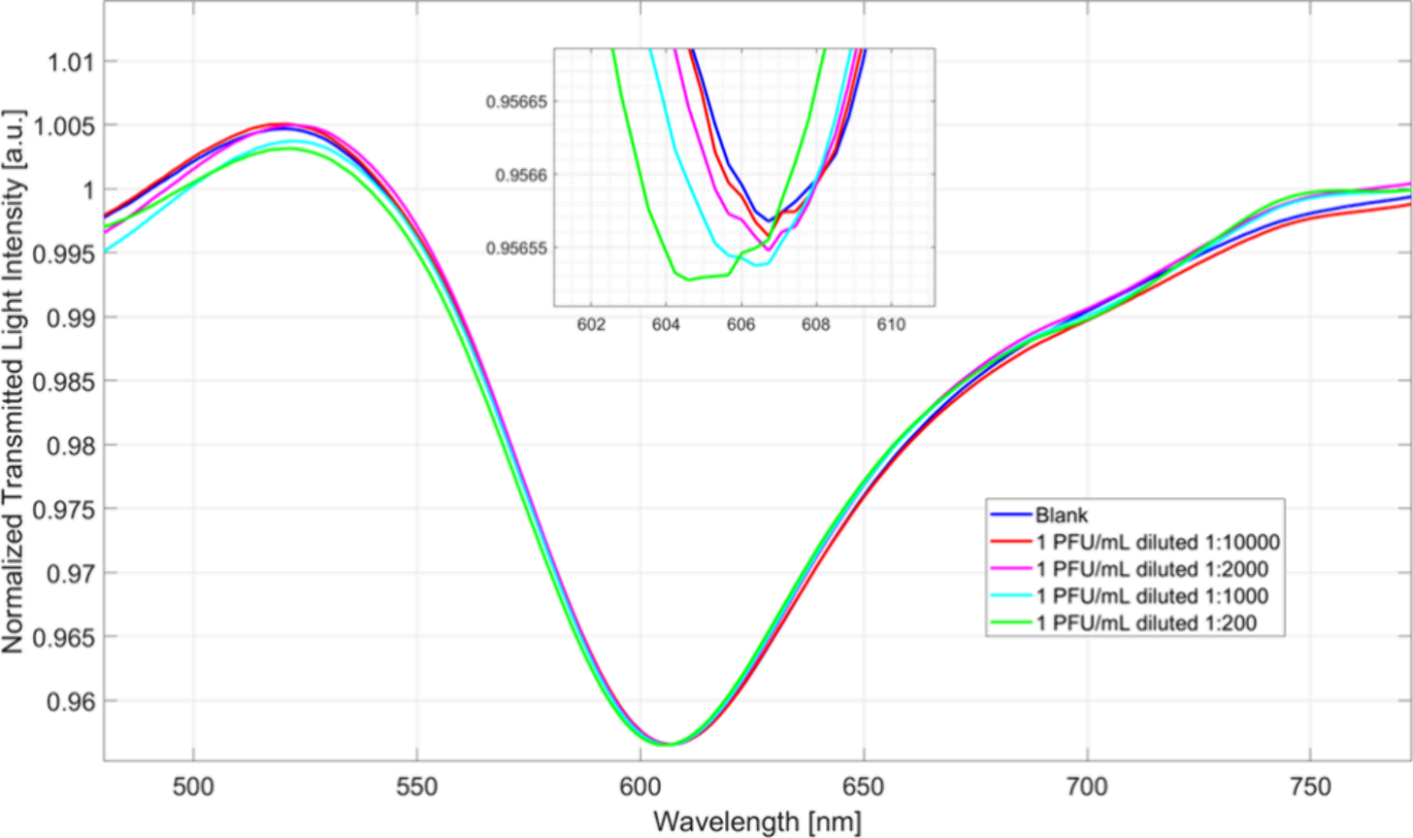
SPR spectra obtained using the POCT with lysed TOSV 1 PFU/mL solutions at different dilution ratios in PBS (1:10000, 1:2000, 1:1000, 1:200).

#### Selectivity Tests

The selective performance of the SPR-POF biochip was evaluated by testing the POCT with various viral agents to verify its ability to recognize TOSV selectively. Three viruses, i.e., HSV-1, MeV, and SFNV, were selected as potential interferent viruses. Specifically, these viruses can cause symptoms that are very similar to those of the target virus (TOSV).

Each interfering virus was tested at 500 PFU/mL, whereas the target analyte (TOSV) was tested at 50 PFU/mL. The corresponding variations in the resonance wavelength across the samples are shown in Figure 5.

**Figure 5:**
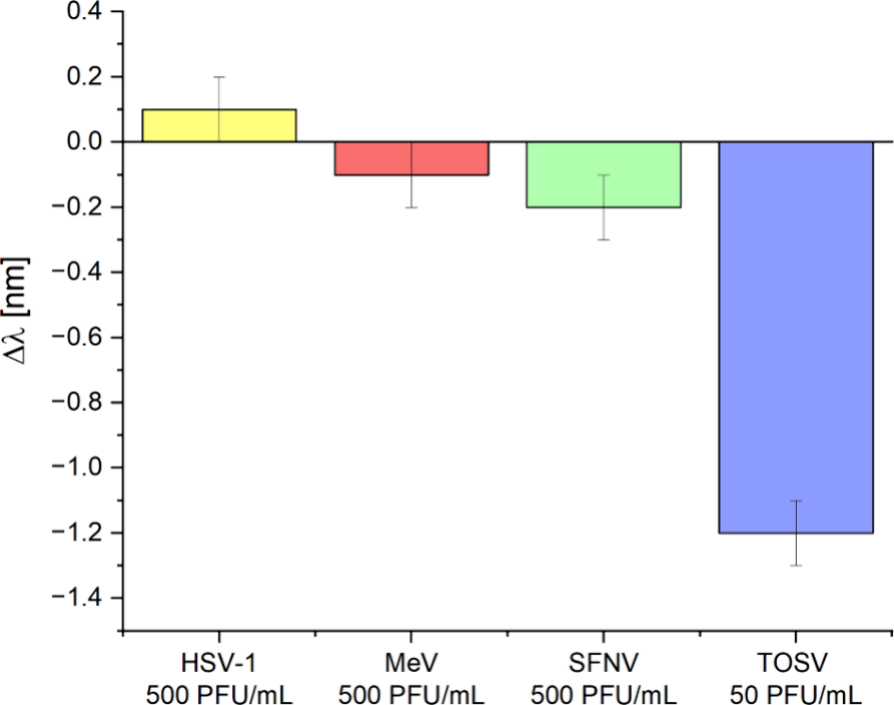
Resonance wavelength shifts (Δλ) obtained by testing the SPR-POF biochip with three interfering viruses, HSV-1, MeV, and SFNV (500 PFU/mL), and with the target TOSV (50 PFU/mL).

The results clearly demonstrate that the POCT exhibits high selectivity against TOSV. The interfering viruses did not produce significant changes in the resonance wavelength. Notably, no detectable signal was observed for SFNV, despite its close structural similarity to TOSV. The observed lack of cross-reactivity with SFNV is most likely attributable to epitope-level sequence divergence in the NP between TOSV and SFNV.^30^ Although both viruses belong to the *Phlebovirus* genus and share overall NP structural homology, amino acid variability in surface-exposed regions of NP can lead to differential antibody recognition. The monoclonal antibody used in this study is therefore expected to preferentially recognize TOSV-specific epitopes that are not fully conserved in SFNV. Consequently, cross-reactivity with SFNV is effectively avoided. In contrast, exposure to TOSV caused a marked shift in the resonance wavelength, despite the significantly lower viral concentration.

#### Testing of Real Samples of Cerebrospinal Fluid

To evaluate the performance of the POCT in a real matrix, the biosensor was tested using four clinical cerebrospinal fluid samples, which were propagated in vitro on Vero cells, as described in paragraph Sample preparation and measurement protocol of Materials and Methods. As representative cases, two samples (named sample 1 and sample 2) with known viral titers of 10^5^ and 10^4^ PFU/mL were reported. Additionally, a negative sample relative to TOSV-negative cerebrospinal fluid was also analyzed. The SPR spectra achieved in these tests are shown in Figure 6A and 6C, respectively.

**Figure 6:**
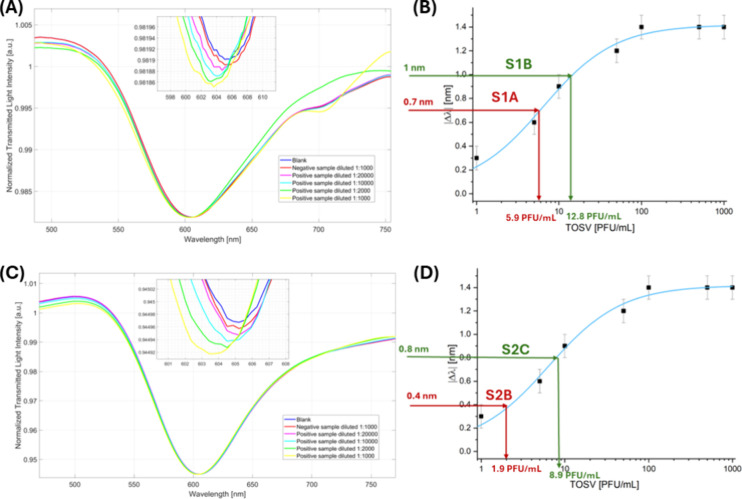
(A) SPR spectra correlated with negative and positive (10^5^ titrated) TOSV samples in CSF diluted with PBS at different dilution factors. Blank refers to the supernatant of uninfected cells. (B) Estimation of TOSV concentration from the dose/response curve of the CSF sample at different dilutions. (C) SPR spectra correlated with negative and positive (10^4^ titrated) TOSV samples in CSF diluted with PBS at different dilution factors. Blank refers to the supernatant of uninfected cells. (D) Estimation of TOSV concentration from the dose/response curve of the CSF sample at different dilutions.

The corresponding resonance wavelength shifts for each dilution were then correlated with the dose–response curve (calibration curve) shown in Figure 3B, thereby enabling the estimation of the TOSV concentration in the original sample.

More specifically, for each measured wavelength shift (|Δλ|), the associated viral concentration was derived from the calibration curve, as shown in Figure 6B and 6D and summarized in Table S2 in the Supporting Information. The final concentration of TOSV in the undiluted viral isolate was calculated by multiplying the estimated concentration of the diluted sample by the corresponding dilution factor.

According to the experimental data reported in Table S2 in the Supporting Information, the sample concentration was approximately 1.25 × 10^5^ PFU/mL. This estimate was validated using two independent measurements (samples S1A and S1B) at different dilutions, yielding consistent results. Samples S1C and S1D, however, could not be used for quantitative analysis because their responses fell within the saturation region of the biosensor dose–response curve.

A second positive sample with an initial titer of 10^4^ PFU/mL was tested using the same dilution factors in PBS, as shown in Figure 6C. In this case, as summarized in Table S3 in the Supporting Information, reliable TOSV concentration values were obtained for samples S2B and S2C (Figure 6B), estimating a TOSV concentration of 1.85 × 10^4^ PFU/mL. However, samples S2A and S2D were not used to estimate the TOSV concentration because they were near the LOD and the sensor’s saturation point, respectively.

#### qPCR of Real Samples of Cerebrospinal Fluid

To corroborate the previously obtained data, four viral cultures derived from the TOSV-positive cerebrospinal fluid samples were analyzed by qPCR. Specifically, the expression of the *NP* and *M* genes was assessed. Amplification reactions producing a sigmoidal curve with a threshold cycle (Ct) value ≤ 33 were classified as positive, those with values between 34 and 40 were considered inconclusive (N/A), and reactions with Ct values >40 were designated as negative.

qPCR amplification of both NP and M genes was reliably observed only in the most concentrated samples, corresponding to viral concentrations between 50 and 100 PFU/mL, and in the most concentrated sample (sample 1). In contrast, lower concentrations yielded inconclusive amplification results (Ct > 33). The deviation from the ideal Ct scaling observed at low viral concentrations can be attributed to stochastic sampling effects near the assay detection limit, as well as variability in reverse transcription and amplification efficiency, which become increasingly significant at low template copy numbers. This test is essential to demonstrate the proposed biosensor’s performance relative to the gold standard (qPCR). ELISA-based assays were considered less suitable due to the limited availability of standardized kits for TOSV NP detection and the well-known risk of serological cross-reactivity associated with phlebovirus infections. Even if the sensing strategies are based on different principles and targets, this comparison confirmed that the SPR-POF biosensor enabled detection down to 0.78 PFU/mL, highlighting an analytical sensitivity approximately 3 orders of magnitude higher than qPCR under the adopted experimental conditions, as shown in Tables S4 and S5 in the Supporting Information. These results demonstrate the capability of the proposed POCT platform to identify TOSV at concentrations substantially below the qPCR detection range, consistent with the analytical performance previously observed for similar SPR-POF biosensing platforms developed by our group.^10^

## Conclusions

In this work, a POCT for the rapid detection of TOSV by identifying its NP was developed and tested. The device showed high sensitivity, with a LOD of 0.78 PFU/mL, as well as excellent selectivity against other clinically relevant neurotropic viruses.

The experimental results have been compared with those obtained by qPCR, since it represents the current clinical gold standard for TOSV detection in cerebrospinal fluid samples. With the experimental conditions adopted, the POCT showed a sensitivity approximately 3 orders of magnitude higher than qPCR.

Further studies are needed to evaluate its direct applicability to pure CSF clinical samples, which are difficult to obtain. The results highlight the strong potential of the proposed technology as a rapid, inexpensive, and easy-to-use POC diagnostic tool. Implementing this strategy could greatly enhance clinical management, differential diagnosis, and surveillance of TOSV infections, particularly in endemic regions and during emergencies.

## Supplementary Material



## Data Availability

All data generated or analyzed during this study are included in this published article [and its Supporting Information files], however raw data will be available from the corresponding author on reasonable request.

## Notes

Figure S1. SPR spectra of the functionalization process. The spectra were obtained in pure water, before and after the functionalization steps. The inset highlights the red shift in the resonance wavelength of the functionalized surface with respect to the bare surface. Table S1. Langmuir parameters relative to the fit of experimental values obtained in PBS shown in Figure 3B. Table S2. Summary of the SPR-POF biochip response to a real 10^5^ titrated CFS sample tested at different dilution factors and estimation of TOSV concentration from the binding isotherm. Table S3. Summary of SPR-POF biochip response of real 10^4^ titrated CFS sample tested at different dilution factors and estimation of TOSV concentration from the binding isotherm. Table S4. Ct values obtained for Sample 1, with *N* representing the TOSV NP gene and *M* corresponding to the TOSV M segment. Table S5. Ct values obtained for Samples 2, 3, and 4. *NP* corresponds to the nucleocapsid gene, and *M* to the M segment of TOSV. (PDF)
