# Supplementary material for: Toscana Virus Diagnosis via a Point-of-Care Tool Based on Disposable Optical Fiber Biosensors
Source: Anal Chem. 2026 Jun 15;98(25):18901–9. doi: 10.1021/acs.analchem.6c01827 (PMC13325457; doi:10.1021/acs.analchem.6c01827)
Supplement: Supplementary file 1 [file ac6c01827_si_001.pdf]

Supplementary Information

Toscana virus Diagnosis via a Point-of-Care tool based on disposable optical fiber biosensors

Federica Passeggio[1,2], Carla Zannella[3], Francesco Arcadio[1], Chiara Marzano[1], Rosalba Pitruzzella[1], Luigi Zeni[1], Bianca Maria Nastri[3], Anna De Filippis[3], Sven Reiche[4], Cécile Baronti[5], Bruno Coutard[5], Maria Grazia Cusi[6], Massimiliano Galdiero[3,7], Giuseppe Portella[2,*], Nunzio Cennamo[1,*]

[1] Department of Engineering, University of Campania Luigi Vanvitelli, 81031 Aversa, Italy
[2]Department of Translational Medical Sciences, University of Naples Federico II, 80131 Naples, Italy
[3] Department of Woman, Child and General and Specialized Surgery, University of Campania Luigi Vanvitelli, 80138 Naples, Italy
[4] Department of Experimental Animal Facilities and Biorisk Management, Friedrich-Loeffler-Institut, 17493 Greifswald – Insel Riems, Germany
[5]Unité des Virus Émergents, Aix-Marseille Univ, Università di Corsica, IRD 190, Inserm 1207, IRBA, Marseille, France.
[6]Department of Medical Biotechnologies, University of Siena, 53100 Siena, Italy
[7]UOC Virology and Microbiology, University Hospital Luigi Vanvitelli, 80138 Naples, Italy

*corresponding authors: portella@unina.it; nunzio.cennamo@unicampania.it

Table of Contents

Figure S1 SPR spectra of the functionalization process

...…………………………………………………... S3

Table S1 Langmuir fitting parameters

..……………………………..……………………………………..... S3

Table S2 Summary of the SPR-POF biochip response to a real 10^5 titrated CFS sample ……….... S3

Table S3 Summary of SPR-POF biochip response of real 10^4 titrated CFS sample

……..……..…... S4

Table S4 Ct values obtained for Sample 1

……………………………………………………….……….... S4

Table S5 Ct values obtained for Samples 2, 3, and 4

………………………..…………………………..... S5

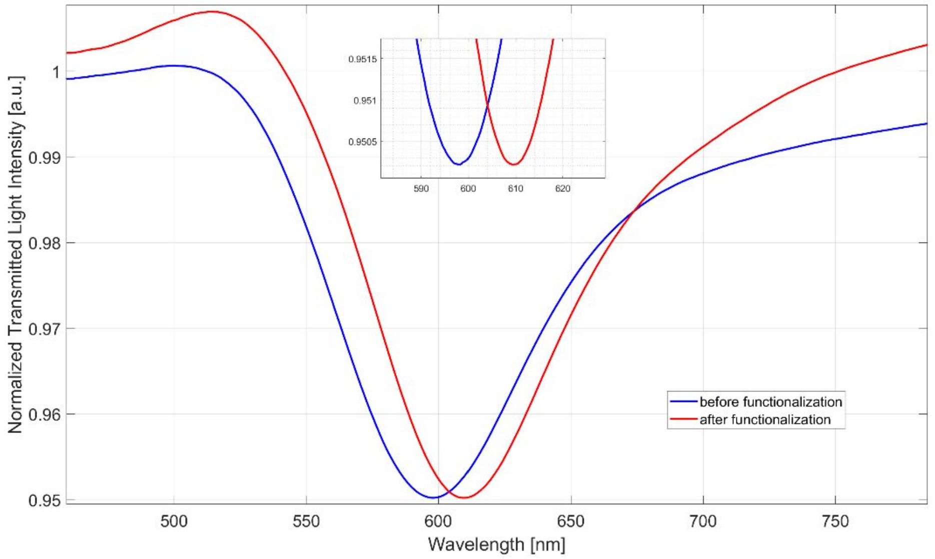

Figure S1. SPR spectra of the functionalization process. The spectra were obtained in pure water, before and after the functionalization steps. The inset highlights the red shift in the resonance wavelength of the functionalized surface with respect to the bare surface.

Table S1. Langmuir parameters relative to the fit of experimental values obtained in PBS shown in Figure 3B.

$\|\lambda_0\|$ [nm]		$\|\Delta\lambda_{max}\|$ [nm]		K [PFU/mL]		Statistics	
Value	St. Error	Value	St. Error	Value	St. Error	X^2	R^2
0.04	0.05	1.42	0.03	6.44	1.07	0.35	0.99

Table S2. Summary of the SPR-POF biochip response to a real 10^5 titrated CFS sample tested at different dilution factors and estimation of TOSV concentration from the binding isotherm.

**Diluted positive sample label**	**Dilution factor**	**$\|\Delta\lambda\|$ [nm]**	**Estimated TOSV concentration of the diluted sample [PFU/mL]**	**Estimated TOSV concentration of the**

				sample [PFU/mL]
S1A	1:20000	0.7	5.9	118000 (1.2 x 10^5)
S1B	1:10000	1	12.8	128000 (1.3 x 10^5)
S1C	1:2000	1.5	near saturation value	-
S1D	1:1000	1.5	near saturation value	-

Table S3. Summary of SPR-POF biochip response of real 10^4 titrated CFS sample tested at different dilution factors and estimation of TOSV concentration from the binding isotherm.

Diluted positive sample label	Dilution factor	$\|\Delta\lambda\|$ [nm]	Estimated TOSV concentration of the diluted sample [PFU/mL]	Estimated TOSV concentration of the sample [PFU/mL]
S2A	1:20000	0	near LOD value	-
S2B	1:10000	0.4	1.9	19000 (1.9 x 10^4)
S2C	1:2000	0.8	8.9	17800 (1.8 x 10^4)
S2D	1:1000	1.3	near saturation value	-

Table S4. Ct values obtained for Sample 1, with *N* representing the TOSV NP gene and *M* corresponding to the TOSV M segment. Ct values are reported as mean ± standard deviation (SD) of three independent technical replicates. N/A: inconclusive; ND: not determined. Ct values correspond to target gene amplification (TOSV *NP* and *M*) and are reported as raw Ct values. GAPDH was included as an internal reference gene for RNA quality control and was not used for ΔCt normalization in the analysis presented in this study.

**Dilution tested (PFU/mL)**	**Ct obtained in qPCR** Sample 1			
	NP	***SD***	***M***	***SD***
100	25.4	0.3	28.7	0.4
50	27.3	0.2	30.5	0.5
10	N/A	ND	N/A	ND

5	N/A	ND	N/A	ND

Table S5. Ct values obtained for Samples 2, 3, and 4. *NP* corresponds to the nucleocapsid gene, and *M* to the M segment of TOSV. Ct values are reported as mean ± standard deviation of three independent technical replicates. Ct values correspond to target gene amplification (TOSV *NP* and *M*) and are reported as raw Ct values. GAPDH was included as an internal reference gene for RNA quality control and was not used for ΔCt normalization in the analysis presented in this study.

**Dilution tested (PFU/mL)**	**Ct obtained in qPCR** Sample 2		**Ct obtained in qPCR** Sample 3		**Ct obtained in qPCR** Sample 4	
	NP	***M***	***NP***	***M***	***NP***	***M***
10	N/A	N/A	N/A	N/A	N/A	N/A
5	N/A	N/A	N/A	N/A	N/A	N/A
1	N/A	N/A	N/A	N/A	N/A	N/A
0.5	N/A	N/A	N/A	N/A	N/A	N/A
